# Comparing the Postpartum Quality of Life Between Six to Eight Weeks and Twelve to Fourteen Weeks After Delivery in Iran

**DOI:** 10.5812/ircmj.16985

**Published:** 2014-07-05

**Authors:** Nosrat Bahrami, Zahra Karimian, Somayeh Bahrami, Nahid Bolbolhaghighi

**Affiliations:** 1 Department of Midwifery, Dezful University of Medical Sciences, Dezful, IR Iran; 2 Department of Reproductive Health, Shahroud University of Medical Sciences, Shahroud, IR Iran; 3 Department of Statistics, Dezful University of Medical Sciences, Dezful, IR Iran; 4 School of Nursing and Midwifery, Shahroud University of Medical Sciences, Shahroud, IR Iran

**Keywords:** Quality of Life, Postnatal, Depression

## Abstract

**Background:**

Women during the postpartum period experience many physiological, psychological, and social changes. Quality of life (QOL) is a sense of well-being and arises from satisfaction or dissatisfaction with various aspects of life including health, employment, socioeconomic state, psychological-emotional state, and family. Moreover, QOL is an important criteria for assessing healthcare system.

**Objectives:**

The purpose of this study was to compare the postpartum QOL between six to eight and 12 to 14 weeks after delivery in women referred to public health centers in Dezful City, Iran, in 2011.

**Materials and Methods:**

This study was a longitudinal study. The study participants were 150 postpartum women referred to public health centers. Quota method was used for sampling. Data collection tools in this study were demographic questionnaire, Edinburgh Postnatal Depression Scale (EPDS), short form health survey questionnaire (SF-36), and Specific Quality of Life after Delivery Questionnaire. Data were analyzed using SPSS.

**Results:**

The results showed that the mean scores of various dimensions of the SF-36 were significantly higher at 12 to 14 weeks than at six to eight weeks (P < 0.001). The postpartum mean depression score was significantly higher at six to eight weeks than at 12 to 14 weeks (P < 0.001). The mean score of QOL questionnaires at 12 to 14 weeks were increased in all dimensions in comparison with six to eight weeks; however, this increase was significant only in dimension of the mother's feelings toward herself, her husband, and others (P < 0.001).

**Conclusions:**

Because enormous changes develop in postpartum women, we suggest supportive measures for mother by her mother-in-law, family, and caregivers to improve the QOL and health status of the mother and her child.

## 1. Background

Quality of life (QOL) is a multidimensional concept that affects performance of the individual in physical, psychological, social, and spiritual aspects of life and can be affected by political, cultural, economic, and spiritual beliefs (1). World Health Organization (WHO) definition of QOL includes six dimensions: physical health; psychological-emotional status; level of independence; social relationships; spiritual dimensions; and environmental situation. In a general definition, QOL is the effect of the physical and social environment on individual and ontological and emotional reactions to this environment (2).

According to the WHO definition, QOL is the people's perception of their position in life in context of culture and value systems that they live and in relation to the goals, expectations, communication, needs, and beliefs. Determining life concept and measuring QOL is important for public health policy, research, evaluation, and clinical decision-making (3). QOL is a sense of well-being and arises from satisfaction or dissatisfaction with various aspects of life, and includes areas such as health, employment, socioeconomic status, psychological-emotional status, and family (4).

QOL is an important indicator of the quality of healthcare (5). As a part of disease control programs (6), QOL should be measured from different perspectives. Assessment of QOL is important for making social as well as medical and clinical decisions (7). Several factors affect the QOL such as delivery and physical, mental, and social factors (8). Women during the postpartum period experience many physiological, social, and psychological changes; therefore, they need more attention during this period (9). In recent years, experts have become aware of the importance of the healthcare in delivery (10) because only a few women were healthy in postpartum studies (11).

Less attention is drawn toward the mothers’ problems such as fatigue, back pain, itching in cesarean incision, perineal pain, and hemorrhoids and women have to adapt to these problems. In addition, these problems have a significant effect on the physical, emotional, and social health, breastfeeding, relationships with family, community, and childcare, and housework (12). This problem affects both mothers' and children’s health (13). One of the objectives of the WHO was promoting maternal health status and reducing morbidity and mortality rates by 2010. According to WHO statistics, the highest maternal mortality and morbidity are seen in postpartum period (14). Studies in Australia and the United States showed that more than 50% of women had mental health problems in the first year after delivery (15, 16). A study conducted in the United States showed a significant decrease in postpartum QOL score in the physical-emotional dimension (17).

The postpartum period is an important time for a mother, her newborn, and her family. Women problems may increase during the postpartum period (18, 19) and these changes might affect mother’s well-being (20). Additionally, women reports considerable limitations in their abilities at work as well as at home even up to six months after delivery (21).

Assessing QOL in this period will allow a woman to make a self-evaluation of her own postpartum situation and will assist healthcare providers with further promotion of women’s and infants’ health (22). Variety of medical, psychological, social, and obstetric factors might affect the QOL after delivery. Mothers’ negative perception of their own health may have a negative effect on their infant-care behaviors. The mothers’ health after childbirth can affect their children’s health (23).

## 2. Objectives

Considering the importance of the postpartum QOL and the limited researches in this area, we aimed to compare postpartum QOL between six to eight and 12 to 14 weeks after delivery in women referred to public health centers in Dezful City, Khuzestan province, Iran, in 2011.

## 3. Materials and Methods

This study was a longitudinal study. The study participants were 150 postpartum women referred to public health centers of Dezful city, Iran. Sample size was calculated by the following formula:

N = (z_1 - α/2_ + z_1 - β_)^2^ (σ_1_^2^ - σ_2_^2^) [(m - 1) × 0.7/d^2^]

Where α = 0.05, z_1 - α/2 _= 1.96, β = 0.20, z_1 - β _= 0.84, d = 0.05, and m = 2.

Women who had delivered a single live child were enrolled in the study if they met the following criteria: postpartum women at six to eight weeks who aged between 18 to 35 years, were literate, and received prenatal care. In addition, women with the following condition were excluded: stillbirth or abnormal birth, difficult delivery, birth weight of less than 2500 g, a history of abortion or any medical illness, medical complications of pregnancy, history of infertility, depression, drug use, and stress, or family problems. Quota sampling method was used for recruiting participants. Following explaining the study protocol and purpose, a written informed consent was signed by the participants. The Ethics Committee of Dezful University of Medical Sciences approved the study protocol. Data collection tools in this study were demographic questionnaire, Edinburgh Postnatal Depression Scale (EPDS), short form health survey questionnaire (SF-36), and Specific Quality of Life after Delivery Questionnaire (SQOLAD).

The EPDS questionnaire was developed to screen postpartum depression. This questionnaire includes ten questions concerning common symptoms of depression and the total sum of the scores is 30 (24). A total score of ten or higher were considered as depression. The SF-36 includes eight dimensions with the sum general score in each dimension ranging from zero and 100; a higher score indicates better health status (25). SQOLAD includes 30 questions in eight different dimensions; the general score in each dimension ranges from zero to 100 with higher scores indicating better health status (26).

The EPDS, SF-36, and SQOLAD were validated using content validity method; reliability of these questionnaires was obtained by test-retest method in which 0.85, 0.81, and 0.78 were obtained for these tests, respectively (26-29). In this study, questionnaires were completed by face-to-face interview at six to eight weeks after delivery. Then we requested the mothers to come back during 12 to 14 weeks after delivery to complete the same questionnaires. Data were analyzed by SPSS (SPSS Inc., Chicago, IL, USA) using paired-samples t test, Chi square, Mann-Whitney U, and Wilcoxon ranked tests. Statistical significance was determined at P < 0.05.

## 4. Results

In this study, the majority of women were homemakers (84%), and the majority of their husbands were self-employed (64.6%). The education level of the majority women and their husbands was high school (68.3% and 62.3%, respectively), and 56.5% had normal vaginal delivery. Other demographic characteristics of subjects are shown in Table 1.

**Table 1.** The Demographic Characteristics of Women Referred to the Dezful Health Center ^a, b^

Group Characteristics	Value	Median	IQR
**Age of Mothers, y**	25.8 ± 6.4	26.5	11.25
**Age of Husbands, y**	32.7 ± 4.6	30.4	6.14
**BMI, kg/m** ^**2**^	24.3 ± 1.5	23.5	4.32
**Occupation**			
Housewife	124 (82.6)	-	-
Employed	26 (17.4)	-	-
**Woman’s Educational Years**			
< 12	98 (65.3)	-	-
> 12	52 (34.7)	-	-
**Spouses’ Educational Years**			
< 12	44 (29.3)	-	-
> 12	106 (70.7)	-	-
**Social-Economical status**			
Good	36 (24)	-	-
Intermediate	114 (76)	-	-
**Fertility Mode of Delivery**			
Vaginal Delivery	90 (60)	-	-
Cesarean Section	60 (40)	-	-
**Gender of Neonate**			
Female	72 (48)	-	-
Male	78 (52)	-	-

^a^ Data are presented as mean ± SD and No. (%).

^b^ Abbreviations: BMI, body mass index; IQR, interquartile range.

**Table 2.** The Short Form Health Survey Scores at Six to Eight Weeks and Twelve to Fourteen Weeks After Delivery ^a^

Dimensions	Score at 6-8 Weeks			Score at 12-14 Weeks			P Value
	Mean ± SD	Median	IQR	Mean ± SD	Median	IQR	
**Bodily Pain**	82.2 ± 11.4	80.6	15.4	89.34 ± 18.74	91.6	23.62	0.009
**General Health**	53.71 ± 31.26	51.5	38.8	56.21 ± 25.22	53.1	31.7	< 0.001
**Vitality**	57.02 ± 18.23	58.4	23.87	65.11 ± 15.74	67.4	21.05	< 0.001
**Physical Functioning**	51.8 ± 18.48	49.78	22.53	59.5 ± 17.31	55.6	24.43	< 0.001
**Role Physical**	31.25 ± 15.87	33.7	19.58	37.45 ± 16.59	36.2	21.9	< 0.001
**Mental Health**	51.42 ± 20.13	48.43	25.7	69.65 ± 22.34	71.9	28.91	< 0.001
**Role Emotional**	39.71 ± 19.71	36.57	26.5	47.51 ± 17.56	50.1	23.6	< 0.001
**Social Functioning**	49.25 ± 20.3	45.3	28.7	57.34 ± 19.24	59.76	26.3	< 0.001

^a^ Abbreviation: IQR, interquartile range.

The mean score of postpartum depression in women at six to eight weeks was significantly higher than their mean score at 12 to 14 weeks (P < 0.001). Various aspects of SF-36 scores in six to eight weeks and 12 to 14 weeks are shown in Table 2. Various aspects of health questionnaire scores were significantly higher in 12 to 14 weeks than in six to eight weeks (P < 0.001). The mean score for the physical performance of women after six to eight weeks was the highest score among different dimensions of SF-36 and showed a very good level of physical performance. The mean scores for empowerment of women at six to eight weeks were the lowest score among different dimensions of the SF-36, which indicated poor quality of empowerment.

Different dimension scores of SQOLAD at 12 to 14 weeks after delivery increased in all dimensions from six to eight weeks postpartum; however, this increase was only significant in mother's feelings toward herself, her husband, and others (P < 0.001). According to the obtained data from this study, scores of SQOLAD were moderate.

## 5. Discussion

The results of this study showed that mean depression score decreased significantly from six to eight weeks to 12 to 14 weeks after delivery. Other studies conducted in Iran have shown similar results (28). Postpartum depression has negative effect on daily function and QOL of both mother and infant. Mothers with depression may be emotionally away from their baby due to physical and psychological stress during labor and baby support can be difficult for them (30, 31).

On the one hand, because postpartum depression is associated with impaired QOL (32, 33), taking care of the mother during pregnancy and postpartum period is necessary. The mean score for the physical performance of women at six to eight weeks after delivery was the highest score among the different dimensions of SF-36, which indicated a very good level of physical performance.

The mean scores for empowerment of women at six to eight weeks after delivery were the lowest scores among the different dimensions of the SF-36 and showed poor quality of empowerment. Similarly, a research conducted in the Netherlands showed that the mean physical function score of postpartum women at six weeks was the highest score (8). On the other hand, studies in Canada indicated that physical function scores were higher in women after delivery, which was contrary to our results (25). The reason for this difference might be participation of pregnant women with more than one delivery in Canadian study; in other words, pregnant women with more than one delivery may be familiar with changes during pregnancy and after delivery and are more likely to improve their physical performance. Research conducted in the United States showed that empowerment scores after delivery were reduced, which is in line with the results of the present study (17).

Considering that fatigue constitutes up to 67% of postpartum symptom, achieving such results is justifiable (34); however, in studies in Canada and the Netherlands, women empowerment scores after delivery were high, which is contrary to our results (8, 25). The cause of this difference might be the participation of pregnant women in prenatal education classes, exercise during pregnancy, provided support from their husbands, and cultural differences. According to our study, the mean QOL scores after delivery was moderate. In research conducted in Turkey, the postpartum QOL was determined as moderate, which is similar to our results. In addition, supporting mother significantly improves the postpartum QOL (35).

The scores of all the different dimensions of SQOLAD were increased from six to eight weeks to 12 to 14 weeks after delivery; however, this increase was only significant in mother's feelings toward herself, her husband, and others. Results of this study showed similar results in women who delivered via cesarean section; however, these results differed from the results of a study on women with vaginal delivery in which they only had increased sense of motherhood toward their child (26). The reason for this difference might be the presence of a large number of women with vaginal delivery and different socioeconomic status in this study. In our study, sampling strategy and inability to generalize finding to the target population were among the study limitation.

According to the results of this study, QOL in postpartum women was moderate. There are emotional, physiological, and social changes after delivery and eight weeks following delivery that will return to the baseline after a long time. We recommend taking protective measures by the mothers-in-law, families, and caregivers to improve QOL and health status of the mothers and their children.

## References

[1] Ioannidis G, Gordon M, Adachi JD (2001). Quality of life in osteoporosis.. Nurs Clin North Am..

[2] Fairclough DL (2002). Design and Analysis of Quality of Life Studies in Clinical Trials..

[3] Frank-Stromborg M, Olsen SJ (2004). Instruments for Clinical Health-care Research..

[4] Sammarco A (2001). Perceived social support, uncertainty, and quality of life of younger breast cancer survivors.. Cancer Nurs..

[5] Wong JG, Cheung EP, Chen EY, Chan RC, Law CW, Lo MS (2005). An instrument to assess mental patients' capacity to appraise and report subjective quality of life.. Qual Life Res..

[6] Dougherty CM, Dewhurst T, Nichol WP, Spertus J (1998). Comparison of three quality of life instruments in stable angina pectoris: Seattle Angina Questionnaire, Short Form Health Survey (SF-36), and Quality of Life Index-Cardiac Version III.. J Clin Epidemiol..

[7] Katz S (1987). The science of quality of life.. J Chronic Diseases..

[8] Jansen AJ, Essink-Bot ML, Duvekot JJ, van Rhenen DJ (2007). Psychometric evaluation of health-related quality of life measures in women after different types of delivery.. J Psychosom Res..

[9] Pillitteri A (2007). Maternal & Child Health Nursing: Care of the Childbearing & Childrearing Family..

[10] Zhou SZ, Wang XL, Wang Y (2009). Design of a questionnaire for evaluating the quality of life of postpartum women (PQOL) in China.. Qual Life Res..

[11] Glazener CM, Abdalla M, Stroud P, Naji S, Templeton A, Russell IT (1995). Postnatal maternal morbidity: extent, causes, prevention and treatment.. Br J Obstet Gynaecol..

[12] Symon A, MacDonald A, Ruta D (2002). Postnatal quality of life assessment: introducing the mother-generated index.. Birth..

[13] Cheng CY, Fowles ER, Walker LO (2006). Continuing education module: postpartum maternal health care in the United States: a critical review.. J Perinat Educ..

[14] Bahadoran B, Abbasi F, Yousefi AR, Kargarfard M (2008). Evaluating the effect of exercise on the postpartum quality of life.. Iran J Nurs Midwifery Res..

[15] Brown S, Lumley J (2000). Physical health problems after childbirth and maternal depression at six to seven months postpartum.. BJOG..

[16] McGovern P, Dowd B, Gjerdingen D, Gross CR, Kenney S, Ukestad L (2006). Postpartum health of employed mothers 5 weeks after childbirth.. Ann Fam Med..

[17] Gjerdingen DK, Center BA (2003). First-time parents' prenatal to postpartum changes in health, and the relation of postpartum health to work and partner characteristics.. J Am Board Fam Pract..

[18] Hammoudeh W, Mataria A, Wick L, Giacaman R (2009). In search of health: quality of life among postpartum Palestinian women.. Expert Rev Pharmacoecon Outcomes Res..

[19] MacArthur C, Winter HR, Bick DE, Knowles H, Lilford R, Henderson C (2002). Effects of redesigned community postnatal care on womens' health 4 months after birth: a cluster randomised controlled trial.. Lancet..

[20] Hill PD, Aldag JC, Hekel B, Riner G, Bloomfield P (2006). Maternal Postpartum Quality of Life Questionnaire.. J Nurs Meas..

[21] Maimburg RD, Vaeth M, Durr J, Hvidman L, Olsen J (2010). Randomised trial of structured antenatal training sessions to improve the birth process.. BJOG..

[22] Bahrami N, Simbar M, Bahrami S (2013). The Effect of Prenatal Education on Mother's Quality of Life during First Year Postpartum among Iranian Women: A Randomized Controlled Trial.. Int J Fertil Steril..

[23] Huang K, Tao F, Liu L, Wu X (2012). Does delivery mode affect women's postpartum quality of life in rural China?. J Clin Nurs..

[24] Logsdon MC, Hutti MH (2006). Readability: an important issue impacting healthcare for women with postpartum depression.. MCN Am J Matern Child Nurs..

[25] Da Costa D, Dritsa M, Rippen N, Lowensteyn I, Khalife S (2006). Health-related quality of life in postpartum depressed women.. Arch Womens Ment Health..

[26] Torkan B, Parsay S, Lamieian M, Kazemnezhad A, Montazeri A (2008). Comparative analysis of life quality in mothers after cesarean section and normal vaginal delivery.. Iran J Nurs Midwifery Res..

[27] Montazeri A, Goshtasebi A, Vahdaninia M, Gandek B (2005). The Short Form Health Survey (SF-36): translation and validation study of the Iranian version.. Qual Life Res..

[28] Montazeri A, Torkan B, Omidvari S (2007). The Edinburgh Postnatal Depression Scale (EPDS): translation and validation study of the Iranian version.. BMC Psychiatry..

[29] Zubaran C, Foresti K, Schumacher MV, Muller LC, Amoretti AL (2009). An assessment of maternal quality of life in the postpartum period in southern Brazil: a comparison of two questionnaires.. Clinics (Sao Paulo)..

[30] Beck CT (2006). Postpartum depression: it isn't just the blues.. Am J Nurs..

[31] Cunningham FG (2005). Williams Obstetrics..

[32] Beusterien KM, Steinwald B, Ware JE, Jr (1996). Usefulness of the SF-36 Health Survey in measuring health outcomes in the depressed elderly.. J Geriatr Psychiatry Neurol..

[33] Boyce PM, Johnstone SJ, Hickey AR, Morris-Yates AD, Harris MG, Strachan T (2000). Functioning and well-being at 24 weeks postpartum of women with postnatal depression.. Arch Womens Ment Health..

[34] Dritsa M, Da Costa D, Dupuis G, Lowensteyn I, Khalife S (2008). Effects of a home-based exercise intervention on fatigue in postpartum depressed women: results of a randomized controlled trial.. Ann Behav Med..

[35] Akyn B, Ege E, Kocodlu D, Demiroren N, Yylmaz S (2009). Quality of life and related factors in women, aged 15-49 in the 12-month post-partum period in Turkey.. J Obstet Gynaecol Res..

